# Contrasting Trends of Surface PM_2.5_, O_3_, and NO_2_ and Their Relationships with Meteorological Parameters in Typical Coastal and Inland Cities in the Yangtze River Delta

**DOI:** 10.3390/ijerph182312471

**Published:** 2021-11-26

**Authors:** Min Lv, Zhanqing Li, Qingfeng Jiang, Tianmeng Chen, Yuying Wang, Anyong Hu, Maureen Cribb, Aling Cai

**Affiliations:** 1 School of Geographic Sciences, Nantong University, Nantong 226007, China; qfjiangz@ntu.edu.cn (Q.J.); ayhu2018@ntu.edu.cn (A.H.); 2 Earth System Science Interdisciplinary Center, Department of Atmospheric and Oceanic Science, University of Maryland, College Park, MD 20740, USA; zhanqing@umd.edu (Z.L.); mcribb@umd.edu (M.C.); 3 State Key Laboratory of Severe Weather, Chinese Academy of Meteorological Sciences, Beijing 100101, China; chentm@cma.gov.cn; 4 Key Laboratory for Aerosol-Cloud-Precipitation of China Meteorological Administration, School of Atmospheric Physics, Nanjing University of Information Science & Technology, Nanjing 210044, China; yuyingwang@nuist.edu.cn; 5 Meteorological Forecast Center, Zhangpu Meteorological Bureau, Zhangzhou 363000, China; 890001@nuist.edu.cn

**Keywords:** PM_2.5_, O_3_, NO_2_, meteorological parameters, geographical locations, Yangtze River Delta

## Abstract

The contrasting trends of surface particulate matter (PM_2.5_), ozone (O_3_), and nitrogen dioxide (NO_2_) and their relationships with meteorological parameters from 2015 to 2019 were investigated in the coastal city of Shanghai (SH) and the inland city of Hefei (HF), located in the Yangtze River Delta (YRD). In both cities, PM_2.5_ declined substantially, while O_3_ and NO_2_ showed peak values during 2017 when the most frequent extreme high-temperature events occurred. Wind speed was correlated most negatively with PM_2.5_ and NO_2_ concentrations, while surface temperature and relative humidity were most closely related to O_3_. All of the studied pollutants were reduced by rainfall scavenging, with the greatest reduction seen in PM_2.5_, followed by NO_2_ and O_3_. By contrast, air pollutants in the two cities were moderately strongly correlated, although PM_2.5_ concentrations were much lower and O_x_ (O_3_ + NO_2_) concentrations were higher in SH. Additionally, complex air pollution hours occurred more frequently in SH. Air pollutant concentrations changed more with wind direction in SH. A more effective washout effect was observed in HF, likely due to the more frequent strong convection and thunderstorms in inland areas. This research suggests pertinent air quality control measures should be designed accordingly for specific geographical locations.

## 1. Introduction

The adverse impacts of ambient air pollution on human health and the ecosystem have been widely recognized [1,2]. At present, serious haze pollution—e.g., fine particulate matter (PM_2.5_) and rebounded groun.cd−level ozone (O_3_) pollution are the most concerning issues in China [3,4]. As one of the most important precursors of PM_2.5_ and O_3_ and key contributors to atmospheric oxidation, nitrogen dioxide (NO_2_) plays key roles on both PM_2.5_ and O_3_ levels in complex air pollution [5,6,7]. For this purpose, obtaining a thorough understanding of the trends of PM_2.5_, O_3_, and NO_2_ is urgently needed.

Concentrations of these air pollutants are notably influenced by both emissions and meteorological conditions [8,9]. Although much effort has been made to tackle air pollutant emissions, severe air pollution events still occur under some stagnant meteorological conditions in China [4]. An increase in the occurrence of O_3_ pollution has been reported in the majority of cities in China, in contrast to decreases in PM_2.5_ pollution levels [10,11]. Additionally, given the complex chemical reactions among primary air pollutants and relatively long life that allows for changing meteorological processes, variable levels of air pollutants can occur [12,13]. To successfully control air pollution, it is thus nontrivial to further identify the roles of distinct meteorological conditions on levels of air pollutants [3,14].

The Yangtze River Delta (YRD), an economically developed and densely populated area, is located on the east coast of China, comprising Shanghai City, Jiangsu Province, Zhejiang Province, and Anhui Province [15]. Owing to rapid urbanization and industrialization, the YRD has experienced poor air quality in recent years. Many studies have investigated air pollution characteristics and their relationships with meteorological conditions in this area [6,16,17]. Most previous studies investigating trends of surface air pollutants and meteorological impacts on them have been mainly carried out in one city or area of the YRD [18,19,20,21,22]. Differences among regions in the YRD and influential factors have received relatively little attention, especially between coastal and inland cities. According to previous studies, atmospheric circulation, dispersion, and deposition can result in systematic differences in air pollutant concentrations between coastal and inland cities only a few hundred kilometers apart [23,24,25]. However, these studies mainly involve short−term observations made in other parts of the country. The different characteristics of air pollutants between coastal and inland areas remain unclear, especially in the YRD. Studies based on simultaneously made long−term observations from coastal and inland cities are thus imperative to understand the impact of different geographical locations on the trends of surface air pollutants and their relationships with meteorological conditions in the YRD.

In this study, we investigated three main air pollutants, i.e., PM_2.5_, O_3_, and NO_2_, in a coastal city and an inland city in the YRD to gain insight into the influence of meteorological conditions on these air pollutants. Based on simultaneously measured long-term datasets of air pollutants and meteorological conditions from surface monitoring stations in Shanghai (SH) and Hefei (HF) from 2015 to 2019, the following issues are addressed: (1) the long-term trends of PM_2.5_, O_3_, and NO_2_ concentrations in coastal and inland cities; (2) the quantitative links between these air pollutant concentrations and meteorological variables in coastal and inland cities; and (3) the disparity in the impact of meteorological conditions in coastal and inland cities on the levels of air pollutants.

The paper is organized as follows: in Section 2, we briefly describe the data measurement and analysis methods. In Section 3, the long-trends of PM_2.5_, O_3_ and NO_2_, the evolution of complex air pollution hours, and the influence of meteorological parameters on PM_2.5_, O_3_, and NO_2_ are analyzed. Section 4 provides the discussion and conclusions.

## 2. Data and Methods

### 2.1. Study Areas

**Figure 1:**
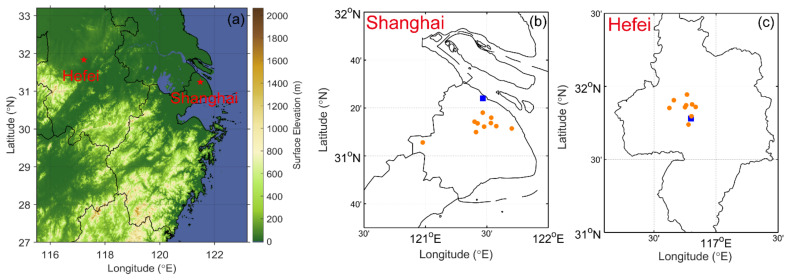
(**a**) Map showing the locations of Hefei and Shanghai in the Yangtze River Delta (YRD) and the locations of air quality (orange circles) and meteorological (blue squares) stations in (**b**) Shanghai and (**c**) Hefei.

Air pollutants, namely, PM_2.5_, O_3_, and NO_2_, in a coastal city (SH) and an inland city (HF) in the YRD for the years 2015 to 2019 are investigated here (Figure 1). SH (31°12′ N, 121°30′ E) is a representative coastal megacity located at the mouth of the YRD region, being a populous urban area and a national center of commerce, trade, and transportation, with the busiest container port in the world. HF (31°52′ N, 117°17′ E) is a typical inland city, about 450 km west of SH, and the capital of Anhui Province, bordering the North China Plain to the north, the Central China Region to the west, and the YRD to the east [26]. HF is generally downwind of prevailing winds from the north and south in cold and warm seasons. In general, the coastal city of SH has better diffusion conditions than the inland city of HF. Air pollution deposition into the sea can largely reduce air pollutants near the coastal area as well [24,27]. Additionally, both cities feature a humid subtropical climate experiencing four distinct seasons. The complex monsoon and synoptic weather may have a substantial impact on air pollution formation and transport in this area [18].

### 2.2. Data and Analysis Methods

Air pollutant and meteorological data were simultaneously collected in SH and HF from 1 January 2015 to 31 December 2019. Real-time, hourly concentrations of air pollutants, including PM_2.5_, O_3_, and NO_2_, at all national air quality monitoring sites were published on an online platform published by the China National Environmental Monitoring Centre (CNEMC), while historical data is not openly available. We used historical data from 1 January 2015 to 31 December 2019 (at https://quotsoft.net/air/ accessed on 15 February 2021) archived by one provider. Three−hourly meteorological data, i.e., air temperature (*T*), dew-point temperature (*T_d_*), atmospheric pressure (*P*), wind speed and direction (*W_s_, W_d_*), rainfall amount (*R*), and horizontal visibility (*VIS*), employed in this study are from the National Climate Data Center (https://www.ncei.noaa.gov/data/global-hourly, accessed on 15 February 2021). Extreme high-temperature events were referred to days in which the daily maximum temperature is above 35 ℃. Relative humidity (*RH*) is calculated from *T* and *T_d_*, based on the Clausius-Clapeyron equation. Wind directions are classified as the following: N, NNE, NE, ENE, E, ESE, SE, SSE, S, SSW, SW, WSW, W, WNW, NW, and NNW. Calm (C) condition is when *W_s_* ≤ 0.2 m/s. All the meteorological parameters were measured 8 times per day (3-h data) for each city. Considering that air pollutant and meteorological data are reported in Beijing Time (BJT) and Universal Time (UTC), respectively, converting UTC to BJT is required, i.e., BJT = UTC + 8 h.

A quality control process was conducted on the data at individual sites to remove problematic data points before calculating average concentrations and parameters. The citywide hourly mean concentrations of PM_2.5_, O_3_, and NO_2_ were calculated by averaging hourly data at all sites in the city, which were used in the analysis, as well as daily, seasonal, and annual mean concentrations. Three-hourly and daily mean meteorological parameters were also employed in this study. The high pollution periods under the joint impact of PM_2.5_ and O_3_ are defined as complex air pollution hours, and the thresholds of hourly mean concentration are 75 μg/m^3^ for PM_2.5_ and 200 μg/m^3^ for O_3_ based on the Ambient Air Quality Standard (GB3095−2012). Days with daily mean *VIS* < 10 km and *RH* < 90% were defined as hazy days according to the observation standard established by the China Meteorological Administration. Otherwise, a non−hazy day was recorded.

Seasons were defined as spring (March, April, and May), summer (June, July, and August), autumn (September, October, and November), and winter (December, January, and February). Regarding descriptive statistics, the least-squares regression method was used to derive the linear trends of the time series of air pollutant concentrations. The Pearson correlation analyses with a two-tailed student’s *t*-test were chosen to evaluate the association between air pollutant concentrations and meteorological variables, which is known as the one of the best methods of measuring the relationship between variables. Due to the ability to provide valuable information about the predictor variables by removing or adding variables and high computation efficiency, for this step, the highest concentrations of O_3_ wise multiple regression was adopted to assess the explained variances of the meteorological parameters on the variations in pollutants concentrations [28,29].

## 3. Results and Discussion

### 3.1. Long-Term Trends of PM_2.5_, O_3_, and NO_2_

**Figure 2:**
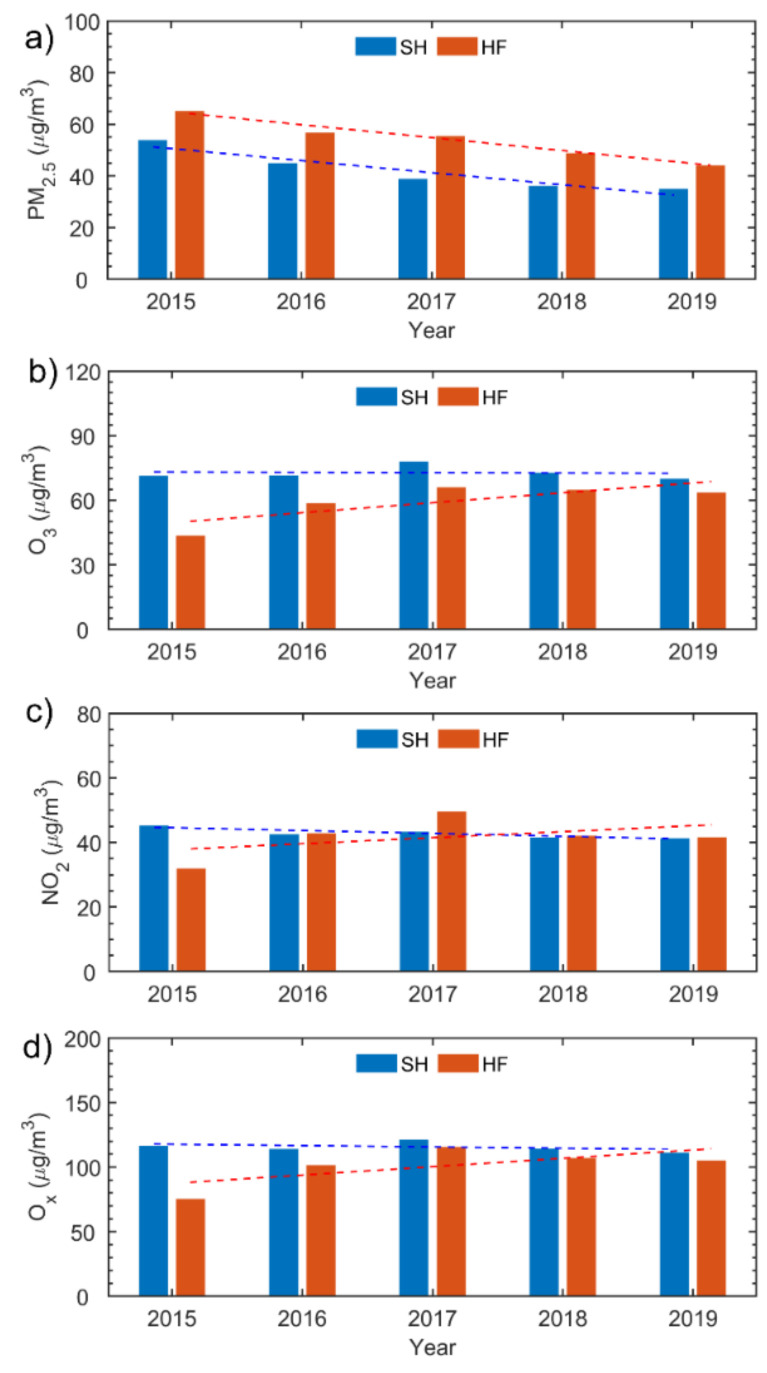
Annual mean concentrations (unit: μg/m^3^) of (**a**) PM_2.5_, (**b**) O_3_, (**c**) NO_2_, and (**d**) O_x_ in Shanghai (SH, blue bars) and Hefei (HF, red bars) over the period 2015–2019. Dashed lines show the long-term trends.

The long-term trends of annual mean concentrations of PM_2.5_, O_3_, and NO_2_ were first investigated (Figure 2). From 2015 to 2019, annual mean concentrations of PM_2.5_ showed significant decreasing trends of −4.7% (*p* < 0.05) in SH and of −5.0% (*p* < 0.01) in HF as a result of the strict regional PM_2.5_ reduction requirements. O_3_ and NO_2_ had fluctuating trends, initially increasing then decreasing slowly around 2017 in both SH and HF. However, O_3_ and NO_2_ showed net decreasing trends of −0.1% and −0.9% in SH, and net increasing trends of 4.6% and 1.9% in HF, respectively, which were insignificant at the 0.01 confidential level (Figure 2 and Table 1). The greatest frequency of the occurrence of extreme high-temperature events (28 days) in 2017 was to blame for the peak annual mean O_3_ and NO_2_ values that year. The trends for different seasons were also calculated (Table 1). Both cities had decreasing trends for PM_2.5_ in all seasons. The trends in summer and autumn in SH (*p* < 0.05) and all seasons except autumn in HF (*p* < 0.1) were statistically significant. As for O_3_, NO_2_, and O_x_, in most seasons, trends slightly decreased (clearly increased) in SH (HF), which were mostly not statistically significant.

Furthermore, trends of the oxidant (O_x_ = O_3_ + NO_2_) were also investigated to evaluate the atmospheric oxidation capacities of distinct cities (Figure 2d). As a comparison, the levels of PM_2.5_ were much lower in SH while the levels of O_x_ were higher in SH than in HF over the entire study period. This suggests that local emissions from vehicle and industrial emissions were more dominant in the gases than PM_2.5_ [19]. Also revealed was the stronger atmospheric oxidation capacity in SH than in HF. The mean concentration of PM_2.5_ in SH was 41.9 μg/m^3^ over the whole study period, much lower than that in HF (54.1 μg/m^3^), which might be due to the favorable diffusion conditions of the coastal location. However, the mean concentration of O_3_ in SH was 72.8 μg/m^3^, approximately 1.22 times that in HF (59.5 μg/m^3^). Differences in NO_2_ between the two cities were relatively smaller. Both coastal and inland PM_2.5_ concentrations still exceeded the minimum safe level (annual mean <35 μg/m^3^) for residential areas according to ambient air quality standards [30]. Overall, the rates of decline of air pollutant concentrations have slowed down in both cities since 2017. This implies that improving air quality in the YRD remains a challenge.

The correlation between SH and HF daily mean air pollutants was further investigated. Moderately strong correlations between daily mean concentrations in SH and HF were obtained, with Pearson’s correlation coefficients of 0.58 (*p* < 0.01), 0.63 (*p* < 0.01), and 0.61 (*p* < 0.01) for PM_2.5_, O_3_, and NO_2_, respectively. These results suggest that not only the local emissions but also the coordinated regional emissions are crucial in making air pollutant control policies.

### 3.2. Evolution of Complex Air Pollution Hours

**Figure 3:**
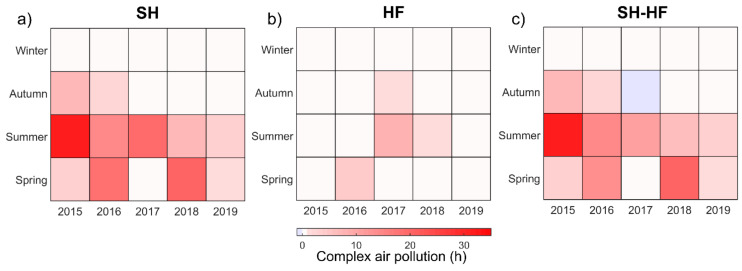
Joint histograms of seasons and years for complex air pollution hours for (**a**) SH, (**b**) HF and (**c**) SH–HF. SH–HF denotes the differences of complex air pollution hours between SH and HF. Abbreviations: SH—Shanghai; HF—Hefei.

Figure 3 showed the evolution of complex air pollution hours with mean PM_2.5_ concentration exceeding 75 μg/m^3^ and O_3_ exceeding 200 μg/m^3^ simultaneously. In general, both cities had decreasing trends for the occurrence of complex air pollution over the period 2015–2019, although sometimes rebounded. The complex air pollution had a seasonal pattern, peaking in summer followed by spring and autumn. No complex polluted hour was found in winter. Distinct differences can be seen between each city. The complex air pollution in SH was worse with 127 h in 40 days than that of 14 h in 4 days in HF. Besides, the complex air pollution hours in SH are higher in most seasons except autumn in 2017 during the study period. This is likely due to hourly PM_2.5_ and O_3_ accumulation caused by the sea—land breeze convergences in SH [31].

### 3.3. Influence of Meteorological Parameters on PM_2.5_, O_3_, and NO_2_

#### 3.3.1. Overview of Correlations between Air Pollutants and Meteorological Parameters

The influence of meteorological parameters on concentrations of PM_2.5_, O_3_, and NO_2_ was quantified using the Pearson correlation analysis (Table 2). Calculated were the correlation coefficients between daily mean values of six meteorological parameters and three air pollutants in different seasons. Regarding similarities, *W_s_* was most negatively correlated with PM_2.5_ and NO_2_ concentrations in all seasons in the two cities, indicating its important role in the dispersion of air pollutants. *R* was most related to PM_2.5_ due to wet deposition by heavy rain, while the relationship with NO_2_ was not significant. The influence of different rainfall categories on air pollutants is investigated in Section 3.3.3. *T* was weakly correlated with PM_2.5_ and NO_2_ concentrations in almost all seasons.

In contrast with PM_2.5_ and NO_2_, *T*, and *RH* (followed by *W_s_*) were the most closely correlated with O_3_. The O_3_ correlations with *T* were positive due to accelerated O_3_ production under high-temperature conditions accelerating photochemical reaction rates, with strong correlations in winter, autumn, and summer. Significant negative correlations between O_3_ concentrations and *RH* were found throughout the year in both cities due to the crucial role water vapor played in decreasing photochemical ozone production by affecting solar ultraviolet radiation [32]. Warm, dry weather is thus more conducive to O_3_ formation than cool, wet weather. The impact of *W_s_* on O_3_ concentrations was complex, showing weaker correlations in all seasons, respectively. This likely resulted from the simultaneous diffusion and vertical mixing effect. Normally, it’s conducive to the build-up of O_3_ formation with stronger vertical mixing and weaker diffusion, however, weaker vertical mixing and stronger diffusion decreased O_3_ concentrations [33]. Section 3.3.2 comprehensively analyzes the relationships between air pollutants and *W_s_*/*W_d_*.

Concerning differences in the meteorological influence on air pollutants between both cities, *W_s_* and *W_d_* had stronger relationships with all air pollutants in SH than in HF. *T* had a weaker relationship with O_3_ in SH. In general, these results are primarily attributed to different meteorological and diffusion conditions experienced by coastal and inland areas.

The explained variance of six meteorological parameters upon the daily variability of PM_2.5_, O_3_, and NO_2_ in different seasons was calculated using a step-wise multiple linear regression method (Table 3). The six meteorological factors together can explain higher variances of summertime and autumntime daily levels of PM_2.5_ (O_3_, NO_2_) in SH and HF, respectively. This suggests that meteorological factors play essential roles in the daily fluctuations of pollutants in summer. Note that the explained variances were generally higher in summer and autumn than in other seasons. Moreover, the explained variances of all air pollutants derived in SH were significantly higher than those in HF in all seasons except for O_3_ in spring and summer. This implies the stronger influence of meteorological parameters in the coastal area.

#### 3.3.2. PM_2.5_, O_3_, and NO_2_ Concentrations for Different Wind Directions and Speeds

**Figure 4:**
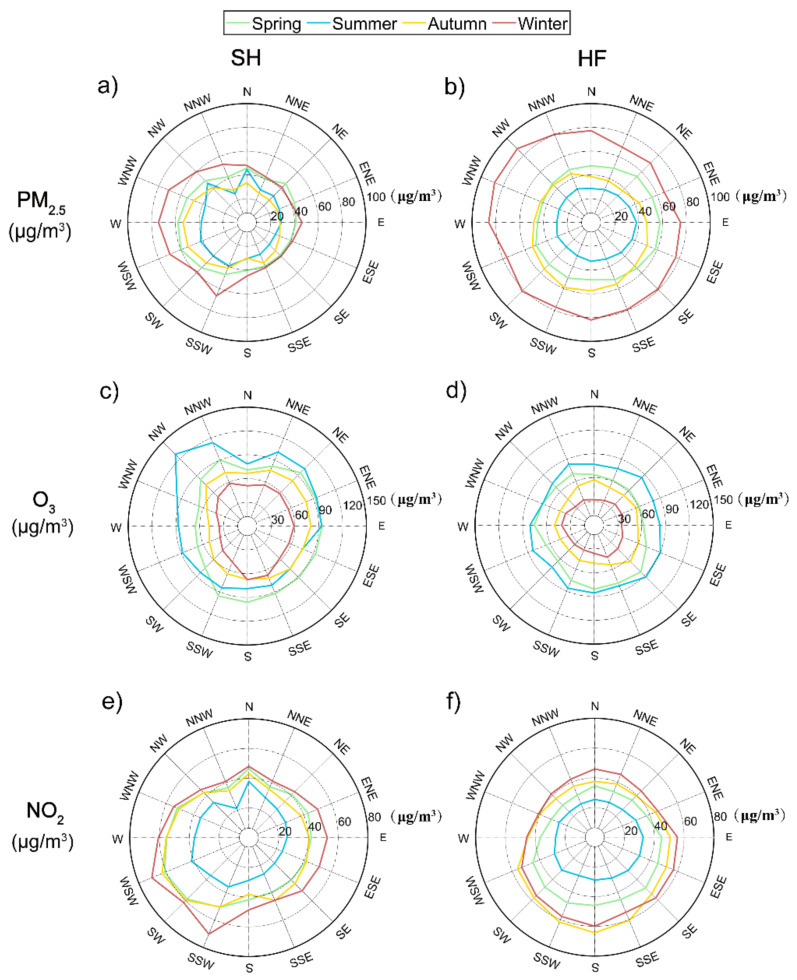
Distributions of seasonal mean concentrations of (**a**,**b**) PM_2.5_, (**c**,**d**) O_3_, and (**e**,**f**) NO_2_ in Shanghai (SH, left panels) and Hefei (HF, right panels). The numbers in each panel show the pollutant concentrations (unit: μg/m^3^).

To better illustrate the influence of wind direction on pollutants, Figure 4 shows seasonal mean concentrations of PM_2.5_, O_3_, and NO_2_ for different wind directions. In general, all air pollutants were distributed more evenly in HF in all seasons than in SH. PM_2.5_ and NO_2_ concentrations in SH were the highest when the wind came from the westerly direction (i.e., W, WSW, and SSW), followed by northerly winds in most seasons, consistent with a previous study [20,30]. This suggests the contribution of transported air pollutants to air pollution episodes in SH was from the west and the north, while the air from the east and south sea was much cleaner with lower emissions. In all seasons, NW winds in SH led to the highest O_3_ concentrations, followed by NE winds. O_3_ concentrations varied little in other wind directions.

By contrast, in HF, peak values of PM_2.5_ concentration were associated with NW, SE and SSW winds, and O_3_ and NO_2_ maximum concentrations were associated with SE winds. However, in all seasons, *W_d_* did not play an important role in changing the concentrations of the three air pollutants in HF as it did in SH. The highest concentrations of PM_2.5_ and NO_2_ occurred in winter, and the highest concentrations of O_3_ occurred in summer for all directions in HF, which demonstrated again the positive correlation between O_3_ and temperature.

Seasonal mean concentrations of PM_2.5_, O_3_, and NO_2_ under calm conditions were also examined, along with the ratios PM_2.5_/CO, O_3_/CO, and NO_2_/CO as indicators of air pollutant secondary formation to primary emissions (Table 4). PM_2.5_ and NO_2_ concentrations were higher, and O_3_ concentrations were much lower under calm conditions than under windy conditions in both cities, i.e., calm conditions were favorable for the accumulation of PM_2.5_ and NO_2_ but unfavorable for O_3_ formation in both coastal and inland cities. As a comparison, the concentrations of PM_2.5_ under calm conditions were lower, and O_3_ concentrations were much higher in SH than in HF in almost all seasons. Three possible reasons are: (1) There was much lower (higher) PM_2.5_ (O_3_ precursors) emissions in SH than in HF according to the Emission Inventory for China (MEIC v1.3, http://meicmodel.org, accessed on 12 August 2021) [34,35]; and (2) Lower PM_2.5_/CO and higher O_3_/CO ratios were found in SH than in HF, revealing weakened PM_2.5_ formation and enhanced O_3_ formation from primary emissions in SH under calm conditions; and (3) Sea−breeze was noticeable resulting in a cycling pattern under the calm wind condition while was not significant under the strong wind condition of prevailing winds in SH [36].

**Figure 5:**
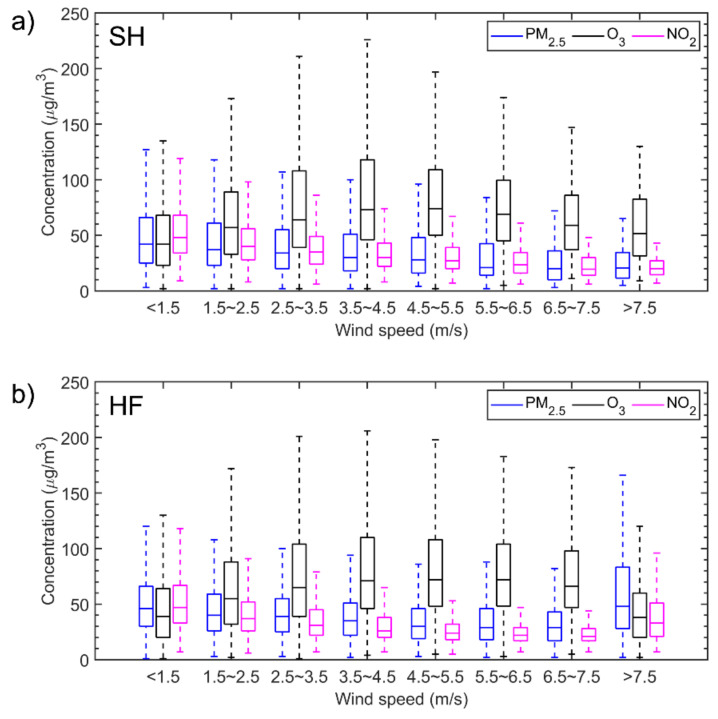
Box and whiskers plot of hourly mean concentrations of PM_2.5_, O_3_, and NO_2_ in each category of wind speed in (**a**) Shanghai (SH) and (**b**) Hefei (HF).

Figure 5 shows hourly mean concentrations of PM_2.5_, O_3_, and NO_2_ in each category of wind speed in SH and HF. Both cities show similar relationships between pollutant concentrations and wind speed. In general, for wind speeds below 7.5 m/s, PM_2.5_ and NO_2_ concentrations decreased as wind speeds increased. This is because high winds tend to disperse air pollutants and dilute PM_2.5_ and NO_2_ concentrations, while stagnant conditions and light winds allow them to build up and become more concentrated. When wind speeds exceeded 7.5 m/s in HF, regional transport might have played a greater role than air dispersal. Concentrations of O_3_ increased to a peak value when wind speeds were between 3.5 and 4.5 m/s, then gradually decreased. These results are mainly ascribed to two simultaneous effects. Higher wind speeds increase air turbulence and facilitate the vertical mixing of upper-level O_3_ to the ground. Higher wind speeds also have a diffusion effect, diluting O_3_ concentrations. When wind speeds are lower, the mixing effect of O_3_ concentration is stronger than the diffusion effect. As wind speeds exceed a threshold value, the diffusion effect dominates again [37].

#### 3.3.3. PM_2.5_, O_3_, and NO_2_ Concentrations for Different Rainfall Categories

**Figure 6:**
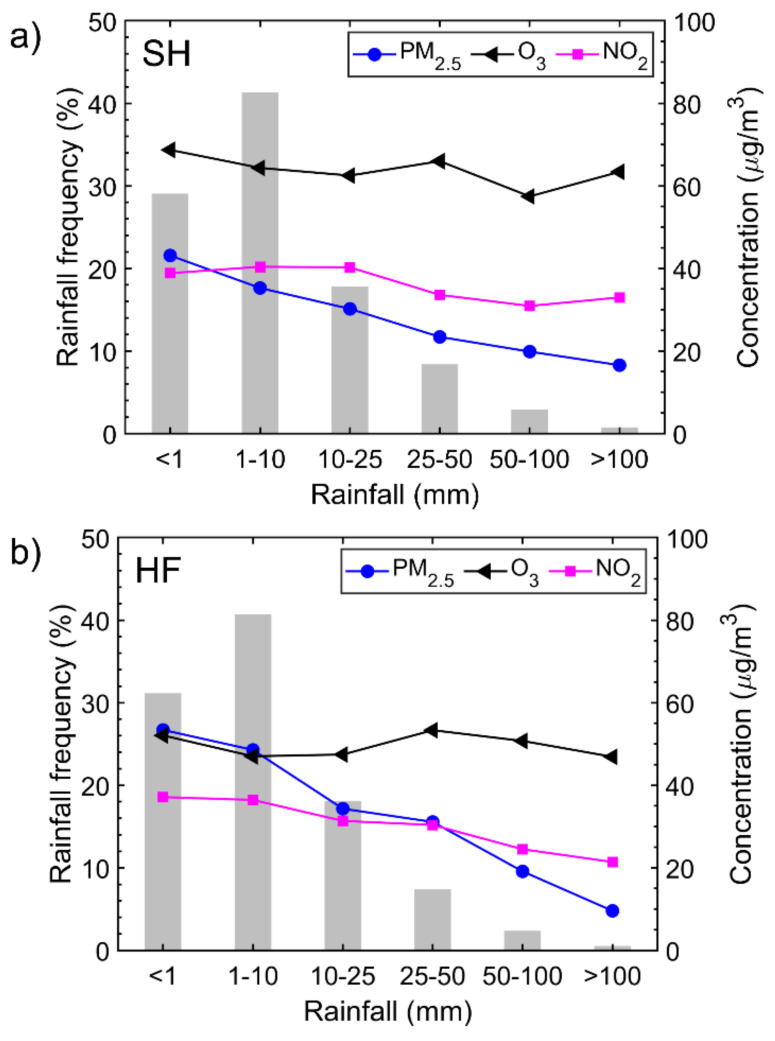
Daily rainfall frequency statistics (gray bars) and average concentrations of PM_2.5_, O_3_, and NO_2_ (colored curves) for each rainfall intensity category in (**a**) Shanghai (SH) and (**b**) Hefei (HF).

Like dispersion and transportation, wet deposition plays a substantial role in modifying air pollutants. Examined next is the influence of daily rainfall scavenging on changes in PM_2.5_, O_3_, and NO_2_ concentrations (Figure 6). At both sites, concentrations of PM_2.5_ were greatly reduced. However, the reduction in O_3_ and NO_2_ (gaseous pollutants) was lower than that of PM_2.5_. The relative effect of rainfall washout on air pollutant concentrations is estimated to be PM_2.5_ > NO_2_ > O_3._ An interesting phenomenon occurred for O_3_ and NO_2_. Their concentrations tended to increase somewhat under rainy conditions. This is likely due to vertical mixing, bringing down a certain amount of O_3_ and NO_2_ from the upper layers of the atmosphere during convective rain activity and thunderstorms [38,39,40,41,42]. Surface NO_2_ concentrations can also be enhanced by lightning-generated NO_2_ during convective rain events [43,44,45]. Rainfall frequency distributions in the two cities were similar, with the highest frequency occurring when the daily rainfall intensity was 1–10 mm. The washout effect for PM_2.5_ and NO_2_ was more effective in HF than in SH, likely due to the greater frequency of strong convection and thunderstorms in inland areas than in coastal areas [46,47]. Concerning O_3_, the washout effect was limited in both cities. Different patterns in O_3_ concentration occurred in SH and HF when the daily rainfall intensity exceeded 25 mm.

#### 3.3.4. PM_2.5_, O_3_, and NO_2_ Concentrations on Hazy and Non−hazy Days

**Figure 7:**
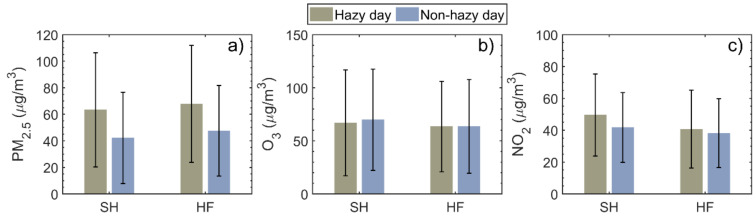
Daily mean concentrations of (**a**) PM_2.5_, (**b**) O_3_, and (**c**) NO_2_ on hazy days (grey bars) and non-hazy days (blue bars) in Shanghai (SH) and Hefei (HF). Error bars denote the standard deviations.

Figure 7 shows daily mean concentrations of PM_2.5_, O_3_, and NO_2_ on hazy days and non−hazy days, calculated using long-term observational data from 2015 to 2019. PM_2.5_ concentrations were higher on hazy days in both cities, i.e., 63.4 and 67.8 μg/m^3^ in SH and HF, respectively, than on non-hazy days, i.e., 42.2 and 47.6 μg/m^3^ in SH and HF, respectively. This amounts to an increase in PM_2.5_ concentration of 50.2% and 42.4% in SH and HF, respectively, mainly due to weakened surface winds, high *RH*, and a low PBL, promoting the accumulation of PM_2.5_ and hygroscopic growth on hazy days. Note that PM_2.5_ concentrations on 34.9% (SH) and 34.4% (HF) of hazy days were greater than 75 μg/m^3^ (the standard for a polluted day), indicating that hazy days remain a major air pollution problem in both coastal and inland cities. Similar results were found for NO_2_, but the differences in NO_2_ concentrations between hazy and non-hazy days were much smaller.

By contrast, O_3_ concentrations were 4.3% and 0.3% higher on non−hazy days than on hazy days in SH and HF, respectively. The suppression of photochemical reactions resulting from reduced sunshine on hazy days, particularly in SH, likely explains this. Higher levels of PM_2.5_ and lower levels of O_3_ and NO_2_ were found in HF compared with SH on both hazy and non−hazy days. These results are consistent with the annual and seasonal trends shown in Figure 2 and Figure 3.

## 4. Conclusions

HF represents a typical inland city located about 450 km west of the typical coastal city of SH, making it a useful model for understanding the influence of different locations on air pollutants in the YRD region. In this study, the contrasting trends of surface PM_2.5_, O_3_, and NO_2_ and their relationships with meteorological parameters in SH and HF were investigated based on surface air pollutant and meteorological datasets from 2015 to 2019. We provide the following conclusions:

In both cities, significant decreasing trends were observed for PM_2.5_, while O_3_ and NO_2_ fluctuated with turning points during 2017 when the most frequent extreme high−temperature events occurred. The rate of decrease in air pollutants slowed in both cities, demonstrating the challenge of persistent reduction in air pollution. Compared with HF, PM_2.5_ concentrations were much lower, O_x_ (O_3_+NO_2_) levels were higher, and the complex air pollution was worse in SH. The correlations of air pollutants between SH and HF were 0.58 (*p* < 0.01), 0.63 (*p* < 0.01), and 0.61 (*p* < 0.01) for PM_2.5,_ O_3_, and NO_2_, respectively, indicating that approximately 60% of time both cities are affected by similar atmospheric conditions due to common regional meteorology.

Considerably different correlations between air pollutants and meteorological parameters were observed, given the diversity of meteorological conditions in both cities. In both cities, *W_s_* was negatively correlated with PM_2.5_ and NO_2_ concentrations, followed by *T* and *R*. Most closely related to O_3_ were *T* (positive correlation) and *RH* (negative correlation), followed by *W_s_*. Compared with HF, *W_s_* and *W_d_* had stronger relationships with all air pollutants while *T* had a weaker relationship with O_3_ in SH, likely due to different sea−land meteorological and diffusion conditions experienced by coastal and inland areas. The six meteorological factors together can explain 68.7% (51.7%, 72.4%) and 56.6% (67.5%, 43.1%) of the variances of summertime daily levels of PM_2.5_ (O_3_, NO_2_) in SH and HF, respectively. Summertime correlation coefficients and explained variances were generally higher than those in other seasons.

Air pollutant concentrations changed more with *W_d_* possibly due to the limit imposed by the shoreline in SH than in HF, where *W_d_* did not play as much of a role in changing air pollutant concentrations. Westerly winds led to the highest PM_2.5_ and NO_2_ concentrations, while NW winds were associated with the highest O_3_ concentrations in SH. In both cities, PM_2.5_ and NO_2_ concentrations showed decreasing trends as a function of *W_s_* under most conditions. O_3_ concentrations increased to a peak value, then gradually decreased. Windless conditions were favorable for PM_2.5_ and NO_2_ but adverse to O_3_ formation in both coastal and inland cities. PM_2.5_ (O_3_) concentrations were lower (higher) in SH than in HF under calm conditions.

All air pollutant concentrations were reduced by rainfall scavenging, with the greatest reduction seen in PM_2.5_, followed by NO_2_ and O_3_. A more effective washout effect was observed in HF, mostly because of the more frequent strong convection and thunderstorms in this inland area. Interestingly, O_3_ and NO_2_ concentrations tended to somewhat increase under rainy conditions in both cities, likely due to convection and lightning, respectively.

A similar increase in PM_2.5_ and NO_2_ concentrations occurred on hazy days compared to non−hazy days. However, O_3_ had higher concentrations on non−hazy days, likely due to the suppression of photochemical reactions resulting from reduced sunshine in the presence of haze. HF had higher levels of PM_2.5_ and lower levels of O_3_ and NO_2_ compared with SH on both hazy and non−hazy days. Further studies of air pollution at coastal and inland sites in other regions of China, as well as the detailed investigations of specific events to learn more about the physical processes leading to the observed differences between coastal and inland cities, are needed to obtain a deeper, more comprehensive understanding of the nationwide air quality problem.

## Figures and Tables

**Table 1:** Annual and seasonal linear trends of air pollutant concentrations from 2015–2019 (unit: μg/m^3^/yr) in Shanghai (SH) and Hefei (HF).

Pollutant	City	Annual	Spring	Summer	Autumn	Winter
PM_2.5_	SH	−4.7 (−8.6) *	−3.1 (−6.0)	−3.8 (−9.3) *	−4.4 (−9.4) *	−5.5 (−7.9)
	HF	−5.0 (−7.7) **	−5.2 (−8.6) **	−3.9 (−9.4) **	−4.1 (−7.0)	−9.1 (−9.7) *
O_3_	SH	−0.1 (−0.2)	0.7 (0.9)	−0.8 (−0.9)	−1.2 (−1.6)	−0.3 (−0.6)
	HF	4.6 (10.5)	6.4 (15.4)	8.0 (13.4) *	2.8 (5.8) **	−0.3 (−0.9)
NO_2_	SH	−0.9 (−2.0) *	−0.4 (−0.9)	−0.9 (−3.0)	−0.7 (−1.6) *	−2.3 (−4.2)
	HF	1.9 (5.8)	3.2 (11.7)	−0.4 (−1.3)	2.3 (6.1)	−1.8 (−4.3)
O_x_	SH	−1.0 (−0.9)	0.3 (0.2)	−1.7 (−1.5)	−2.0 (−1.6)	−2.6 (−2.5)
	HF	6.5 (8.6)	9.6 (14.0)	7.6 (8.8)	5.1 (6.0)	−2.1 (−2.8)

Values in brackets are the trends in units of %/yr. The ** asterisks represent *p* < 0.01, and the * asterisk represents *p* < 0.05, based on the two−tailed Student’s test. Abbreviations: SH—Shanghai; HF—Hefei.

**Table 2:** Correlation coefficients from linear regression relationships between daily mean PM_2.5_, O_3_, and NO_2_ concentrations and meteorological parameters, i.e., temperature (*T)*, relative humidity (*RH)*, pressure (*P)*, wind speed (*W_s_*), wind direction (*W_d_*)*,* and rainfall (*R*), in different seasons in Shanghai (SH) and Hefei (HF). The correlation coefficients for the confidence levels of 0.05 and 0.01 are ±0.20 and ±0.25, respectively, given that the number of samples is much greater than 100 for all correlations. Significance values at *p* < 0.05 are shaded.

Pollutant	Season	City	*T*	*RH*	*P*	*W_s_*	*W_d_*	*R*
PM_2.5_	Spring	SH	−0.04	−0.13	−0.08	−0.22	0.30	−0.30
		HF	0.16	−0.01	−0.11	−0.25	0.15	−0.33
	Summer	SH	0.03	0.03	−0.06	−0.29	0.17	−0.16
		HF	−0.12	0.14	0.08	−0.27	−0.15	−0.12
	Autumn	SH	0.01	−0.18	0.01	−0.42	0.25	−0.14
		HF	0.02	−0.20	0.18	−0.32	−0.17	−0.23
	Winter	SH	−0.23	0.03	0.08	−0.31	0.29	−0.28
		HF	−0.15	−0.13	0.06	−0.21	0.09	−0.19
O_3_	Spring	SH	−0.08	−0.23	0.10	0.28	−0.24	0.15
		HF	0.03	−0.33	0.04	0.29	−0.08	0.17
	Summer	SH	0.35	−0.52	−0.04	0.14	−0.10	−0.16
		HF	0.53	−0.40	−0.23	0.06	0.07	−0.05
	Autumn	SH	0.07	−0.54	0.17	−0.28	0.17	−0.05
		HF	0.24	−0.68	0.06	−0.12	−0.14	−0.22
	Winter	SH	0.52	−0.35	−0.39	0.13	−0.20	−0.05
		HF	0.66	−0.35	−0.02	−0.06	−0.18	−0.05
NO_2_	Spring	SH	0.12	−0.02	−0.12	−0.55	0.16	−0.15
		HF	0.19	−0.26	−0.02	−0.41	−0.07	−0.17
	Summer	SH	−0.07	−0.04	0.01	−0.54	0.21	−0.02
		HF	0.06	−0.33	0.06	−0.34	−0.05	−0.15
	Autumn	SH	−0.11	0.03	−0.02	−0.61	0.38	0.02
		HF	−0.06	−0.29	0.33	−0.51	−0.10	−0.05
	Winter	SH	−0.46	0.06	0.30	−0.58	0.24	−0.20
		HF	−0.17	−0.45	0.06	−0.34	−0.00	−0.24

**Table 3:** Explained variance of six meteorological parameters upon the daily variability of PM_2.5_, O_3_, and NO_2_ in different seasons in Shanghai (SH) and Hefei (HF) (unit: %).

Pollutant	City	Spring	Summer	Autumn	Winter
PM_2.5_	SH	56.5	68.7	68.5	49.7
	HF	46.5	56.6	52.4	42.9
O_3_	SH	48.8	51.7	51.7	34.2
	HF	59.2	67.5	41.8	30.2
NO_2_	SH	51.9	72.4	51.8	41.4
	HF	49.0	43.1	44.7	37.9

**Table 4:** Seasonal mean concentrations of PM_2.5_, O_3_, and NO_2_ under calm conditions in Shanghai (SH) and Hefei (HF) (unit: μg/m^3^). The values in brackets are the ratios of PM_2.5_, O_3_, and NO_2_ to CO under calm conditions.

Pollutant (Pollutant/CO)	City	Winter	Spring	Summer	Autumn
PM_2.5_(PM_2.5_/CO)	SH	52.7 (62.3)	40.0 (53.3)	48.8 (51.3)	64.5 (53.6)
	HF	60.0 (61.5)	39.7 (45.0)	53.2 (50.8)	80.1 (69.4)
O_3_ (O_3_/CO)	SH	46.5 (67.7)	57.9 (85.6)	40.0 (55.0)	21.9 (24.8)
	HF	37.0 (46.3)	52.3 (66.9)	28.1 (34.1)	21.2(23.3)
NO_2_ (NO_2_/CO)	SH	71.4 (88.2)	43.1 (58.1)	69.2 (79.3)	86.4 (79.1)
	HF	63.3 (66.4)	45.2 (53.5)	68.6 (68.5)	65.7 (58.3)

## Data Availability

The long-term air quality monitoring datasets in SH and HF analyzed during the current study are available from https://quotsoft.net/air/ (accessed on 15 February 2021). The meteorological data were obtained from the US National Centers for Environment Prediction’s Global Data Assimilation System (ftp://arlftp.arlhq.noaa.gov/pub/archives/gdas1, accessed on 15 February 2021).
